# Reproducibility of target volumes generated using uncoached 4-dimensional CT scans for peripheral lung cancer

**DOI:** 10.1186/1748-717X-1-43

**Published:** 2006-11-01

**Authors:** Ylanga G van der Geld, Frank J Lagerwaard, John R van Sörnsen de Koste, Johan P Cuijpers, Ben J Slotman, Suresh Senan

**Affiliations:** 1 Department of Radiation Oncology, VU University medical center, Amsterdam, The Netherlands

## Abstract

**Background:**

4-dimensional CT (4DCT) scans are increasingly used to account for mobility during radiotherapy planning. As variations in respiratory patterns can alter observed motion, with consequent changes in the generated target volumes, we evaluated the reproducibility of 4D target volumes generated during repeat uncoached quiet respiration.

**Methods:**

A retrospective analysis was performed on two successive scans (4DCT1 and 4DCT2) generated at the same scanning session for 26 patients with peripheral lung cancer treated with stereotactic radiotherapy (SRT). The volume and position of planning target volumes (PTV_4DCT1 _and PTV_4DCT2_) contoured on both scans were compared, and a dosimetric analysis performed. A SRT plan optimized for each PTV was sequentially applied to the other PTV, and coverage by the 80% isodose was evaluated. Color intensity projections (CIP) were used to evaluate regions of underdosage.

**Results:**

No significant volumetric differences were observed between the two PTVs (*t*-Test p = 0.60). The average displacement of the center of mass between corresponding PTVs was 1.4 ± 1.0 mm, but differences in position were 2.0 mm or greater in 5 cases (19%). Coverage of both PTVs by the 80% prescription isodose exceeded 90% for all but one patient. For the latter, the prescription isodose covered only 82.5% of PTV_4DCT1_. CIP analysis revealed that the region of underdosage was an end-inspiratory position occupied by the tumor for only 10–20% of the respiratory cycle.

**Conclusion:**

In nearly all patients with stage I lung cancer, the PTV derived from a single uncoached 4DCT achieves dosimetric coverage that is similar to that achieved using two such consecutive scans.

## Background

Organ motion in the thorax confounds imaging, radiotherapy treatment planning and treatment delivery for patients with lung cancer [1]. When free breathing CT scans are used for planning, variable geometric errors can arise with respect to the position, shape, and the volume of the gross tumor volume (GTV) and mobile organs [2-4]. With the use of 4-dimensional CT (4DCT), or respiration-correlated CT scans, volumetric image data can be acquired at many different respiratory states, and the time-resolved 3D CT imaging allows organ motion to be characterized for treatment planning [5-8]. A common technique for 4DCT acquisition involves axial cine acquisition with simultaneous recording of an external respiratory signal with over-sampled CT images, which are then sorted into several bins based on the phase information obtained from the respiratory signal [9]. 4DCT was found to be superior to multiple conventional CT scans for generating internal target volumes (ITV) for stereotactic radiotherapy (SRT) of stage I non-small cell lung cancer (NSCLC) [6].

However, the use of 4DCT scans does not remove all mobility-induced artefacts. A study using phantoms found that gated 4DCT acquisition reduced, but did not eliminate volumetric errors when imaging moving objects [3]. Another study reported residual volumetric differences between a static spherical object scanned helically or with 4DCT while moving, to be within ± 5% [10]. In patients, additional problems can arise if the respiratory pattern is not sufficiently reproducible. This results in missing images at a specific respiratory phase for some table indices, leading to gaps in resorted 4DCT volumes [10,11]. Breathing patterns in patients with lung cancer can be complex and exhibit considerable diversity within the same patient [12]. Routine breath coaching has been proposed as a method to improve regular respiratory motion during radiotherapy [5]. However, delivery of fractionated SRT can take up to 40 minutes, which makes coached respiration not feasible in patients who often have severely compromised lung function. As respiration-gated delivery at our center is only used for stage III tumors, and not for SRT for small peripheral tumors, we derived an ITV that was based upon all mobility observed on two consecutive uncoached 4DCT scans.

The aim of the present study was to assess the reproducibility of the two consecutive PTVs based upon uncoached 4DCT scans that were generated at a single imaging session.

## Methods

The successive planning 4DCT scans of 26 consecutive patients with stage I NSCLC who were treated with SRT were retrospectively analyzed.

### 4DCT scanning procedure

The 4DCT scanning approach used at our center has been reported in detail previously [6-8]. Briefly, patients are immobilized in the supine position with both arms positioned above the head on an adjustable arm support. The Real-Time Position Management system (RPM, Varian medical systems, Palo Alto, CA) uses a lightweight block containing two reflective markers placed on the upper abdomen to record the respiratory signal. 4DCT scans are performed during uncoached quiet respiration on a 16 slice CT scanner (Lightspeed 16 GE Medical Systems, Waukesha, WI). CT scanning commences when the RPM system shows a regular respiratory pattern, and multiple respiratory cycles are observed in order to determine the adequate cine scan duration parameter.

For thoracic scans, 8 contiguous slices of 2.5 mm are generated for a 2 cm total longitudinal coverage per gantry rotation with the scanner operated in axial cine mode. Other scanning parameters include 140 KV, 95 mA and tube rotation set closest to 1/10 of the average breathing cycle time to allow high temporal and spatial resolution. With the scanner couch in static mode, data is acquired for at least the duration of one full respiratory cycle with the addition of one second, after which the couch advances to the next position. Data acquisition ceases during the couch movement, and a full 4DCT scanning procedure of the thorax generally takes about 90 seconds. The radiation exposure from 4DCT acquisition is approximately 6 times the dose from a single conventional helical CT scan [8]. As variations in breathing patterns during uncoached CT scans can lead to variations in the defined target volume, our routine imaging protocol for SRT planning requires that a second 4DCT scan (limited to the tumor region) be performed directly after the first. The additional information was considered crucial as doses of 60 Gy (to the 80% isodose) were delivered in 3 fractions with no intra-fractional imaging.

Advantage4D software (GE Medical Systems, Waukesha, WI) is used to retrospectively sort the acquired images into temporally coherent volumetric datasets. Each reconstructed image is assigned to one of 10 respiratory phases based on the temporal correlation between surface motion of the lightweight block and data acquisition. For each couch position, the reconstructed image with the respiratory phase nearest to the requested phase is selected to form the 3D dataset for a given phase of the breathing cycle. The user can define tolerance values for phase selection, and such phase tolerance is necessary in order to obtain complete volumetric information as images at different couch positions are reconstructed at different respiratory phase samples [10].

### Generating Internal Target Volumes (ITVs)

The clinical SRT delivery for each patient was based on non-gated treatment delivery to an ITV that was derived from the summation of contouring on both 4DCT scans. ITVs were generated using maximum intensity projection (MIP) post-processing of data from each 4DCT scan in Advantage4D software. The MIP images reflect the maximum value of any given pixel intensity over all 10 phases of the 4DCT scan, and contoured MIP images have been shown to correspond well with ITVs derived from manual contouring of the corresponding GTVs in all 10 phases of a 4DCT [13].

A single end-expiratory phase of the 4DCT scan and both MIP datasets were imported into the BrainLab stereotactic planning system (Brainscan v 5.2, BrainLab AG, Heimstetten, Germany), and both MIP scans were co-registered with the single phase bin scan using the fusion software tool in Brainscan. If necessary, adjustments were performed to ensure a proper match of the spinal column. The ITV was derived from contouring the tumor on both MIP scans. A contour encompassing both ITV_4DCT_'s was used to derive the ITV for the clinical plan. An isotropic ITV-PTV margin of 3 mm was added in order to account for residual patient setup inaccuracies on the Novalis Exactrac (Brainscan v 5.2, BrainLab AG, Heimstetten, Germany), which uses an online patient setup verification system. No separate margins were used to account for microscopic tumor extension [14].

### Retrospective comparison of PTVs

Two separate PTVs, namely PTV_4DCT1 _and PTV_4DCT2_, were generated and the volumes and location were compared. For the latter, the displacement of the center of mass of both PTVs in the X (medio-lateral), Y (anterior-posterior), and Z (superior-inferior) direction was assessed using the Brainlab coordinate system.

A dosimetric analysis was performed in order to evaluate the clinical relevance of the variations between PTVs, if present. The clinical treatment plan, which consisted of between 8–12 non-coplanar treatment beams, was sequentially modified by adjusting the beam portals to both PTV_4DCT1 _and PTV_4DCT2_. Stereotactic treatment planning was normalized to the 100% isodose at the isocenter. As for the clinical treatment plan, PTV coverage by the 80% isodose was the goal of treatment planning. Coverage of the PTV_4DCT2 _was evaluated using the plan developed for PTV_4DCT1_, followed by the reverse.

### Color intensity projections (CIP)

In order to obtain temporal information in cases with significant underdosage of the PTV based on a single 4DCT, a composite color intensity projection (CIP) image of the 4DCT data was generated [15]. Briefly, the CIP technique generates color images that represent a projection of the intensity changes in the component images into a single image. Pixels whose intensity changes over the phases of the 4DCT appear in the CIP image as colored pixels, and the hue encodes the percentage of time that tissue is present in each location. CIP was implemented as a plug-in tool to ImageJ that was written in-house. ImageJ (version 1.31v) is a Java based image-processing package, and is free public domain software [16].

### Statistics

The Student paired *t*-test for comparison of volume of the PTV_4DCT1 _and PTV_4DCT2 _was performed using Excel software (Microsoft, Redmond, WA).

## Results

Limited volumetric differences were observed between PTV_4DCT1 _and PTV_4DCT2 _(Student *t*-test p = 0.60), with a difference in volume exceeding 10% in 5 out of 26 cases (19%). However, the differences were greater than 1 cc in only 3 patients (12%). The average displacement of the center of mass between the two PTVs was 1.4 ± 1.0 mm, but the difference between the positions of the centers of mass were 2.0 mm or greater in 5 cases (19%) (Table 1).

**Table 1:** Volumetric and spatial comparison of PTV_4DCT1 _and PTV_4DCT2_

**Pat.**	**PTV_4DCT1 _(cm^3^)**	**PTV_4DCT2 _(cm^3^)**	**PTV_4DCT1 _– PTV_4DCT2 _(cm^3^)**	**PTV_4DCT1 _– PTV_4DCT2 _(%)**	**3D center of mass displacement (mm)**
1	28.1	29.3	1.2	4.2	1.7
2	16.2	16.9	0.7	4.5	1.9
3	39.3	38.4	1.0	-2.5	3.2
4	3.5	4.1	0.6	15.6	1.1
5	20.8	21.2	0.5	2.2	2.0
6	3.0	3.3	0.3	11.3	1.2
7	30.5	29.1	1.5	-4.8	1.3
8	9.2	10.0	0.8	8.5	2.6
9	13.9	13.6	0.3	-1.9	0.4
10	29.6	30.3	0.7	2.4	0.8
11	19.7	21.0	1.3	6.7	0.7
12	67.0	68.3	1.3	1.9	0.8
13	13.5	10.9	2.6	-19.4	3.7
14	21.0	20.7	0.3	-1.3	0.6
15	23.0	21.5	1.5	-6.7	1.0
16	13.8	13.5	0.3	-2.0	0.5
17	22.9	25.3	2.4	10.5	1.3
18	63.0	57.8	5.2	-8.3	3.5
19	21.1	21.2	0.2	0.8	1.4
20	23.5	21.9	1.6	-6.8	0.4
21	15.8	16.0	0.3	1.6	1.2
22	26.2	24.5	1.8	-6.8	1.6
23	10.6	10.1	0.5	-4.3	1.2
24	15.6	17.4	1.8	11.8	1.7
25	63.1	64.1	1.0	1.5	0.3
26	45.7	44.9	0.8	-1.8	0.3
**Mean ± SD**			**1.2 ± 1.1 cm^3^**	**0.7 ± 7.6%**	**1.4 ± 1.0 mm**

### Dosimetric consequences

**Figure 1:**
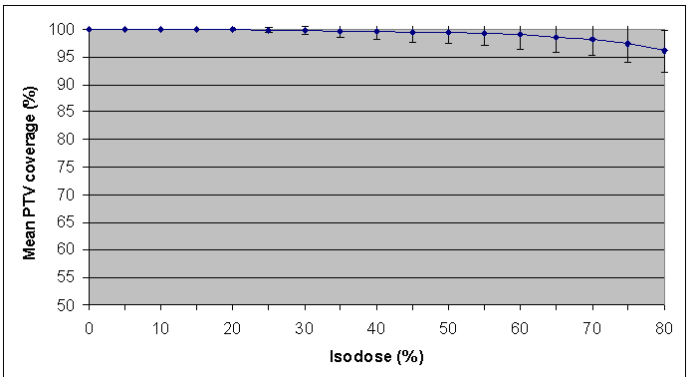
Mean dose-volume histogram for PTV coverage in 26 patients.

**Figure 2:**
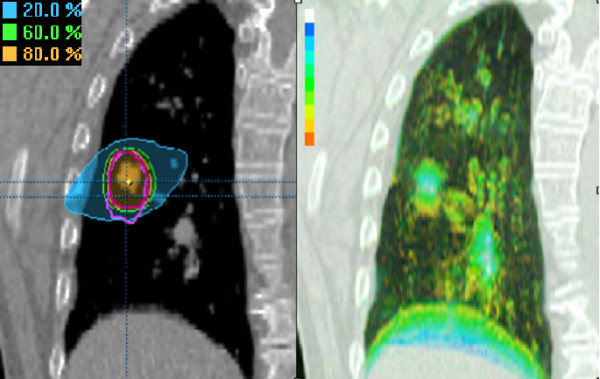
Left panel: Coronal reconstruction of the end-expiration phase bin of patient 13, showing both PTV_4DCT1_(violet contour) and PTV_4DCT2 _(pink contour). Colorwash displays of the 80%, 60% and 20% isodoses show the most caudal area of PTV_4DCT1 _to lie in the 20% isodose area. Right panel: A corresponding color intensity projection in the same patient with the color bar providing time-related positional information (white = 100% presence; blue = 90%; green = 50%; orange = 10%). This indicates that the most caudal PTV region represents a site occupied by the tumor for 10–20% of the respiratory cycle.

Treatment planning was performed using the 8–12 non-coplanar beam setup arrangement used for the clinical treatment of the patient. Beam portals were adjusted to both PTV_4DCT1 _and PTV_4DCT2_, respectively. When satisfactory target coverage was obtained for PTV_4DCT1_, the mean coverage of PTV_4DCT2 _by the resulting 80% prescription isodose was 97.8 ± 2.3%. Alternatively, the mean coverage of PTV_4DCT1 _was 97.0 ± 3.8% when planning was based on coverage of PTV_4DCT2 _(Table 2). The mean dose-volume histogram of PTV coverage in all 26 patients is shown in Figure 1. In all but one patient, the 80% prescription isodose covered >90% of the PTV_4DCT _(Figure 2). In this latter patient (no. 13), only 82.5% of PTV_4DCT1 _was covered when the plan was based upon PTV_4DCT2_. The shift between the center of mass of both 4DCT scans was 3.7 mm in this patient, with a 19% volume difference between both PTV's_4DCT _(Figure 2). The left panel of Figure 2 illustrates the inadequate coverage of the PTV_4DCT1_, and the encompassing 20% isodose suggests a major underdosage at the lower border of the PTV_4DCT1_. The CIP image that was generated from the 4DCT_1 _at the same level, however, illustrates that the inadequate PTV coverage was at an end-inspiratory GTV position, occupied by the tumor for only 10–20% of the respiratory cycle (Figure 2, right panel).

**Table 2:** Percentage of each PTV encompassed by the 80% prescription isodose in a plan optimized for the other PTV.

**Pat.**	**Coverage of PTV_4DCT2 _with planning based on PTV_4DCT1_**	**Coverage of PTV_4DCT1 _with planning based on PTV_4DCT2_**
1	97.8	98.2
2	96.3	97.1
3	98.6	95.2
4	97.8	96.9
5	93.0	99.6
6	96.9	92.9
7	98.9	98.3
8	90.7	93.4
9	98.8	98.9
10	98.9	97.2
11	97.4	100
12	99.9	100
13	97.6	82.5
14	99.2	99.3
15	98.8	95.4
16	99.4	99.6
17	93.9	99.5
18	99.3	91.2
19	99.5	99.8
20	100	98.1
21	99.3	98.1
22	98.8	93.9
23	98.1	97.6
24	94.8	100
25	98.8	98.3
26	100	99.8
**Mean ± SD**	**97.8 ± 2.3%**	**97.0 ± 3.8%**

## Discussion

Individualized margins are required to account for mobility of lung tumors, as no reliable correlation exists between tumor location and mobility [17]. With the use of 4DCT, spatial and temporal information on tumor and normal organ mobility is generated by synchronously recording the respiratory waveform during imaging. The 4DCT process includes two steps, the first of which is the acquisition of several CT images per couch position through the volume of interest, which is followed by resorting of the acquired images into spatiotemporally coherent data sets.

Various approaches for 4DCT acquisition have been reported, one of which is a widely applied technique in clinical practice that uses the axial cine mode [6,8,9]. This approach has been shown to be fairly accurate in phantom studies, but residual motion artifacts arise due to factors such as partial volume projection effects, phase tolerance values for resorting, and irregularities in the breathing pattern of patients [10]. A recent report suggested that the 4DCT system used in our patients fails to generate optimal data in approximately 30% of patients due to image sorting errors [18]. Reasons cited for the latter include the fact that processing of respiratory traces may not accurately reflect the respiratory phase, and that the RPM-software excludes tracing from periods of irregular respiration and Advantage4D disregards the corresponding images. However, with the generation of ITVs using MIP datasets generated from all respiratory phases, image sorting inaccuracies become irrelevant, and remaining differences between 4DCT scans are therefore due to either lacking images of extreme tumor positions as in the described case, or due to breathing irregularities during the CT scanning session.

**Figure 3:**
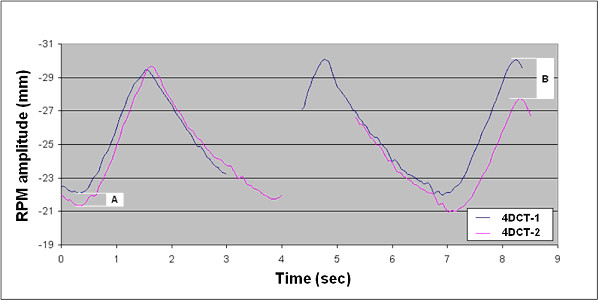
Respiratory waveforms during image acquisition for both 4DCT scans at the level of tumor in patient 13. Images were reconstructed from acquisitions at two consecutive couch positions (table increments of 2 cm) as evidenced by the discontinuous waveforms. The RPM amplitude at the end-expiration tumor position was similar in both 4DCT scans (A), but a larger difference in respiratory amplitude was seen at the end-inspiratory tumor position (B).

In view of concerns about the reproducibility of target volumes derived from uncoached 4DCT scans that were post-processed using the RPM approach, PTV determination in our patients who underwent 3–5 fractions of non-gated curative SRT for lung cancer was based upon data from 2 successive planning 4DCT scans. This analysis of the repeat 4DCT scans in 26 patients revealed that volumetric and spatial differences in PTVs in excess of 10% and 2 mm, respectively, were observed in a fifth of patients. Despite the use of highly conformal stereotactic treatment planning using multiple treatment beams, potential variations in the breathing cycle between CT scanning sessions translated into substantial dosimetric differences in only a single patient. The discrepancy in target volumes for this particular patient may be explained by the 4DCT scanning technique used. We perform axial (cine) scans with table increments of 2 cm, and Figure 3 shows the reconstructed respiratory waveforms during image acquisition at the level of tumor in both 4DCT scans. For this small and mobile tumor, the 4DCT image reconstruction was derived from images that were acquired at two consecutive couch positions as evidenced by the discontinuous waveforms. Although the amplitude at the end-expiration tumor position (most cranial) was similar in both 4DCT scans (A), a larger difference in amplitude was seen during imaging at the end-inspiration tumor position (most caudal), which explains the discrepancy in the caudal border of both PTVs.

Although the absolute underdosage in the most caudal part of the PTV for this fraction of radiation appeared to be important, analysis using the CIP technique revealed that this region of the tumor was present in the area of underdosage for only 10–20% of the time. For the actual stereotactic treatment of this patient, the variation in PTVs was of no consequence as routine clinical plans were based upon ITVs derived from both 4DCT scans. Furthermore, we perform repeat 4DCT scans during the course of SRT [19].

Our results on the reproducibility of uncoached 4DCT scans appear to be even more reassuring in the context of conventionally fractionated 3D conformal radiotherapy. The larger GTV to PTV margins, and certainly also the much lower conformity index of a 3–4 field treatment plan [20], indicates that the variations between ITVs from uncoached 4DCT scans would not have a great impact. Furthermore, previous analysis indicates that incorporating the extremes of respiration-induced tumor movement during conventionally fractionated radiotherapy leads to an overestimation of the PTV [21-24].

Respiratory coaching during 4DCT imaging and treatment delivery has been advocated for patients undergoing gated radiotherapy. This approach is, however, not feasible in patients undergoing SRT as the delivery of a single fraction of non-gated SRT can take more than 30–40 minutes. Furthermore, it is doubtful if patients with medically inoperable stage I NSCLC who generally have severely impaired lung function can tolerate prolonged periods of coached breathing. We will study the variations in respiratory patterns of patients during the delivery of uncoached SRT, and interrupting treatment delivery when amplitude changes in RPM exceed those observed during the 4DCT scan may be one approach to ensure target reproducibility.

## Conclusion

In the majority of patients with stage I lung cancer, the PTV derived from a single uncoached 4DCT achieves similar dosimetric coverage as is achieved using two consecutive 4DCT scans. This indicates that the intra-fractional reproducibility of uncoached 4DCT scans in this patient group is sufficiently reliable in clinical plans based upon target volumes that incorporate all mobility.

## References

[B1] Senan S, Chapet O, Lagerwaard FJ, Ten Haken RK (2004). Defining target volumes for non-small cell lung carcinoma. Semin Radiat Oncol.

[B2] Balter JM, Ten Haken RK, Lawrence TS, Lam KL, Robertson JM (1996). Uncertainties in CT-based radiation therapy treatment planning associated with patient breathing. Int J Radiat Oncol Biol Phys.

[B3] Shen S, Duan J, Fiveash JB, Brezovich IA, Plant BA, Spencer SA, Popple RA, Pareek PN, Bonner JA (2003). Validation of target volume and position in respiratory gated CT planning and treatment. Med Phys.

[B4] Keall PJ, Starkschall G, Shukla H, Forster KM, Ortiz V, Stevens CW, Vedam SS, George R, Guerrero T, Mohan R (2004). Acquiring 4D thoracic CT scans using a multislice helical method. Phys Med Biol.

[B5] Vedam SS, Keall PJ, Kini VR, Mostafavi H, Shukla HP, Mohan R (2003). Acquiring a four-dimensional computed tomography dataset using an external respiratory signal. Phys Med Biol.

[B6] Underberg RWM, Lagerwaard FJ, Cuijpers JP, Slotman BJ, van Sörnsen de Koste JR, Senan S (2004). Four-dimensional CT scans for treatment planning in stereotactic radiotherapy for stage I lung cancer. Int J Radiat Oncol Biol Phys.

[B7] Underberg RWM, Lagerwaard FJ, Slotman BJ, Cuijpers JP, Senan S (2005). Benefit of respiration-gated stereotactic radiotherapy for stage I lung cancer: an analysis of 4DCT datasets. Int J Radiat Oncol Biol Phys.

[B8] van Sörnsen de Koste JR, Senan S, Kleynen CE, Slotman BJ, Lagerwaard FJ (2006). Renal mobility during uncoached quiet respiration: An analysis of 4DCT scans. Int J Radiat Oncol Biol Phys.

[B9] Pan T, Lee TY, Rietzel E, Chen GT (2004). 4D-CT imaging of a volume influenced by respiratory motion on multi-slice CT. Med Phys.

[B10] Rietzel E, Pan T, Chen GT (2005). Four-dimensional computed tomography: image formation and clinical protocol. Med Phys.

[B11] Keall P (2004). 4-dimensional computed tomography imaging and treatment planning. Semin Radiat Oncol.

[B12] Ozhasoglu C, Murhpy MJ (2002). Issues in respiratory motion compensation during external-beam radiotherapy. Int J Radiat Oncol Biol Phys.

[B13] Underberg RW, Lagerwaard FJ, Slotman BJ, Cuijpers JP, Senan S (2005). Use of maximum intensity projections (MIP) for target volume generation in 4DCT scans for lung cancer. Int J Radiat Oncol Biol Phys.

[B14] Potters L, Steinberg M, Rose C, Timmerman R, Ryu S, Hevezi JM, Welsh J, Mehta M, Larson DA, Janjan NA (2004). American Society for Therapeutic Radiology and Oncology and American College of Radiology practice guideline for the performance of stereotactic body radiation therapy. Int J Radiat Oncol Biol Phys.

[B15] Cover KS, Lagerwaard FJ, Senan S (2006). Color intensity projections: A rapid approach for evaluating four-dimensional CT scans in treatment planning. Int J Radiat Oncol Biol Phys.

[B16] ImageJ. http://rsb.info.nih.gov/ij.

[B17] van Sörnsen de Koste JR, Lagerwaard FJ, Nijssen-Visser MR, Graveland WJ, Senan S (2003). Tumor location cannot predict the mobility of lung tumors: a 3D analysis of data generated from multiple CT scans. Int J Radiat Oncol Biol Phys.

[B18] Rietzel E, Chen GT (2006). Improving retrospective sorting of 4D computed tomography data. Med Phys.

[B19] Underberg RW, Lagerwaard FJ, van Tinteren H, Cuijpers JP, Slotman BJ, Senan S (2006). Time trends in target volumes for stage I non-small-cell lung cancer after stereotactic radiotherapy. Int J Radiat Oncol Biol Phys.

[B20] van Sörnsen de Koste JR, Lagerwaard FJ, Schucchard-Schipper RH, Nijssen-Visser MR, Voet PW, Oei SS, Senan S (2001). Dosimetric consequences of tumor mobility in radiotherapy of stage I non-small cell lung cancer – an analysis of data generated using 'slow' CT scans. Radioth Oncol.

[B21] Stroom JC, de Boer HC, Huizenga H, Visser AG (1999). Inclusion of geometrical uncertainties in radiotherapy treatment planning by means of coverage probability. Int J Radiat Oncol Biol Phys.

[B22] Engelsman M, Damen EM, De Jaeger K, van Ingen KM, Mijnheer BJ (2001). The effect of breathing and set-up errors on the cumulative dose to a lung tumor. Int J Radiat Oncol Biol Phys.

[B23] van Herk M, Witte M, van der Geer J, Schneider C, Lebesque JV (2003). Biologic and physical fractionation effects of random geometric errors. Int J Radiat Oncol Biol Phys.

[B24] Engelsman M, Sharp GC, Bortfeld T, Onimaru R, Shirato H (2005). How much margin reduction is possible through gating or breath hold?. Phys Med Biol.

